# Preoperative Osteoporosis Treatment Reduces Stress Shielding in Total Hip Arthroplasty

**DOI:** 10.3390/medsci13010010

**Published:** 2025-01-28

**Authors:** Ryuichi Kanabuchi, Yu Mori, Kazuyoshi Baba, Hidetatsu Tanaka, Yasuaki Kuriyama, Hideki Fukuchi, Hiroki Kawamata, Toshimi Aizawa

**Affiliations:** Department of Orthopaedic Surgery, Tohoku University Graduate School of Medicine, Sendai 980-8574, Japan; ryuichi.kanabuchi.b8@tohoku.ac.jp (R.K.); kazuyoshi.baba.e3@tohoku.ac.jp (K.B.); hidetatsu.tanaka.c7@tohoku.ac.jp (H.T.); yasuaki.kuriyama.b5@tohoku.ac.jp (Y.K.); hf111503@yahoo.co.jp (H.F.); kawamatah1993@gmail.com (H.K.); toshimi.aizawa.a6@tohoku.ac.jp (T.A.)

**Keywords:** total hip arthroplasty, osteoporosis, stress shielding, loosening

## Abstract

**Background:** Total hip arthroplasty (THA) is a widely used surgical intervention for hip osteoarthritis (HOA), with a rising demand driven by an aging population. Osteoporosis is associated with increased risks of bone loss and implant loosening after THA. While medications such as bisphosphonates and denosumab have shown promise in mitigating these risks, the impact of preoperative osteoporosis treatment on postoperative outcomes remains unclear. This study investigates the effect of preoperative osteoporosis treatment intervention on stress shielding and clinical outcomes in THA patients. **Methods:** This retrospective study included 107 patients who underwent cementless THA between April 2019 and March 2022. Patients under 60 years old, with a follow-up period of less than one year, or with prior hip surgery were excluded. Participants were divided into two groups: a treatment group receiving osteoporosis medication preoperatively and a non-treatment group. The outcomes assessed included preoperative bone metabolism markers, Bombelli classification, stress shielding grade, and clinical scores. Statistical analysis was performed using the Mann–Whitney U test and Chi-squared test, with significance set at *p* < 0.05. **Results:** The preoperative osteoporosis treatment intervention rate was 28.9%. Stress shielding grades were significantly lower in the treatment group (*p* = 0.001). However, no significant differences were observed in clinical scores one year postoperatively. **Conclusion:** Preoperative osteoporosis treatment significantly reduced stress shielding incidence, potentially influencing long-term outcomes by preventing bone atrophy-related pain and fractures. Preoperative bone density assessment and osteoporosis treatment intervention are recommended to improve THA outcomes. Larger studies are needed for further validation.

## 1. Introduction

Total hip arthroplasty (THA) is a highly effective surgical intervention for treating hip osteoarthritis (HOA). In Japan, the demand for THA continues to rise annually, with approximately 70,000 procedures performed each year [1]. The increasing demand for THA in Japan is largely driven by the nation’s rapidly aging population, which has led to a higher prevalence of HOA and the need for effective surgical treatments. Several inflammation-related substances associated with joint cartilage damage and their relationship with osteoarthritis have also been discussed [2]. Further discussions on the prevention of osteoarthritis progression remain necessary in the future. The long-term outcomes of cementless femoral stems have shown positive results in both clinical and radiological evaluations, comparable to those of cemented femoral stems. As a result, THA using cementless stems has gained widespread adoption globally [3,4,5].

Nevertheless, several challenges remain to be addressed in THA using cementless stems. One of the major problems is the loosening of the stem due to stress shielding and osteoporosis [6]. The global prevalence of osteoporosis is rising as populations age; however, the low rate of treatment intervention remains a significant concern [7]. The International Society for Clinical Densitometry (ISCD) and the National Osteoporosis Foundation recommends that men over 70 years and women over 65 years have their bone mineral density evaluated before orthopaedic surgery [8]. However, most orthopedic surgeons do not routinely perform bone mineral density testing before joint replacement surgery [9]. Hip fractures in patients with osteoporosis represent a serious issue. Studies using Japan’s nationwide database have highlighted the importance of performing surgery within two days of hospitalization and have reported that THA may offer improved postoperative outcomes compared to bipolar hemiarthroplasty [10,11]. Periprosthetic bone loss following THA is often accompanied by reduced bone density associated with osteoporosis, which is believed to heighten the risk of implant loosening and periprosthetic hip fractures [12,13]. Stress shielding is identified as a cause of implant loosening in THA. Efforts to address this issue include the development of titanium alloy stems with a low Young’s modulus [14,15] and the use of the osteoporosis medication denosumab [16,17], both of which have been reported to help reduce loosening. An interesting research approach has reported that genetic mutations are associated with aseptic loosening following joint arthroplasty [18].

Although there are reports on the effectiveness of denosumab in preventing implant loosening in THA, the efficacy of osteoporosis medications in preventing bone loss or loosening after THA has not been sufficiently investigated. The purpose of this study was to investigate the effect of preoperative osteoporosis treatment intervention on postoperative outcomes in patients who underwent THA for HOA at our hospital.

## 2. Materials and Methods

### 2.1. Patients

This retrospective study adhered to the ethical principles stated in the Declaration of Helsinki and was approved by the ethical review boards of Tohoku University (approval number: No 2021-1-1059). Informed consent was taken from all patients.

This study included patients who underwent cementless THA at our hospital between April 2019 and March 2022. Exclusion criteria were patients under 60 years of age, those with a follow-up period of less than one year, and those with a history of prior hip surgery. A total of 107 patients were selected and categorized into two groups: a treatment group receiving osteoporosis medication prior to surgery and a non-treatment group without any osteoporosis intervention. In the osteoporosis treatment group, the study included cases where the same osteoporosis medication has been continuously administered for over one year.

### 2.2. Clinical Parameters

The evaluation parameters included preoperative bone metabolism markers [bone-specific alkaline phosphatase, tartrate-resistant acid phosphatase, and total procollagen type 1 N-terminal propeptide], the preoperative Bombelli classification, stress shielding grade assessed one year after surgery, and clinical outcomes such as the Japanese Orthopaedic Association (JOA) score, Harris Hip Score (HHS), and the Japanese Orthopaedic Association Hip-Disease Evaluation Questionnaire (JHEQ).

### 2.3. Radiographic Assessments

The Bombelli classification categorizes hip joints into three types—hypertrophic, normal, and atrophic—based on the extent of reparative reactions, such as osteophyte formation and subchondral bone sclerosis, which are characteristic of HOA [19]. Atrophic hip joints exhibit minimal bone proliferation, potentially leading to rapid joint destruction. Hypertrophic hip joints are characterized by excessive osteophyte formation, which can significantly limit the range of motion but may alleviate pain. This classification is essential for predicting a patient’s prognosis. Stress shielding refers to bone atrophy in the proximal region of the fixation caused by differences in the elastic modulus between the femur and the stem implanted into it. This results in an altered load transmission compared to a normal femur. Based on Engh’s criteria [20], the degree of cortical bone atrophy is classified as follows: Grade 0 indicates no bone atrophy; Grade 1 refers to curvature at the osteotomy site with no atrophy in the proximal contact area; Grade 2 involves cortical bone atrophy on the medial side proximal to the lesser trochanter; Grade 3 describes bone atrophy just distal to the lesser trochanter; and Grade 4 indicates bone atrophy extending into the diaphysis.

### 2.4. Statistical Analysis

The results are presented as the mean ± standard deviation. Statistical analysis was conducted using the Mann–Whitney U test and Chi-squared test as appropriate, with a significance level set at *p* < 0.05. Analyses were performed using JMP Software Version 17.2.0 (SAS Institute Japan, Tokyo, Japan).

## 3. Results

**Figure 1:**
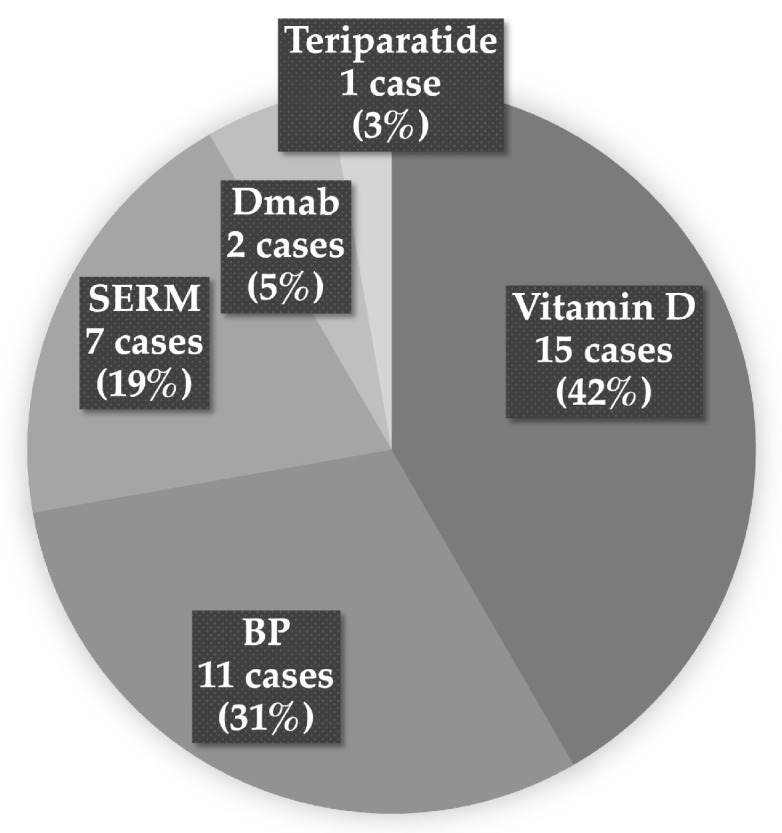
Summary of osteoporosis treatments. BP: Bisphosphonate; SERM: selective estrogen receptor modulator; Dmab: denosumab.

The study population consisted of 12 males and 95 females, with a mean age at the time of surgery of 72.7 years. The rate of osteoporosis treatment intervention prior to THA at our hospital was 28.9%. The distribution of osteoporosis medications used was as follows: vitamin D preparations in 15 cases (42%), bisphosphonates in 11 cases (31%), selective estrogen receptor modulators in 7 cases (19%), denosumab in 2 cases (5%), and teriparatide in 1 case (3%) (Figure 1).

The mean age of the patients in the treatment group was significantly higher than that of the non-treatment group (*p* = 0.033). There was no significant difference in body mass index between the treatment and non-treatment groups (*p* = 0.459). In terms of preoperative bone metabolism markers, bone-specific alkaline phosphatase (bone-type ALP), tartrate-resistant acid phosphatase 5b, and total procollagen type 1 N-terminal propeptide were significantly lower in the treatment group. The Bombelli classification (atrophic type: normotrophic type: hypertrophic type) was 17:19:0 in the treatment group and 33:56:5 in the non-treatment group, with no significant difference observed between the two groups (*p* = 0.129) (Table 1).

Stress shielding grades (grade 0: grade 1: grade 2: grade 3: grade 4) one-year post-surgery were distributed as 12:10:12:2:0 in the treatment group and 6:28:46:11:3 in the non-treatment group, with the treatment group exhibiting significantly lower grades of stress shielding (*p* = 0.001). However, there were no significant differences between the two groups in the JOA score, HHS, and JHEQ one year after surgery (*p* = 0.087, 0.246, and 0.149, respectively) (Table 2).

**Figure 2:**
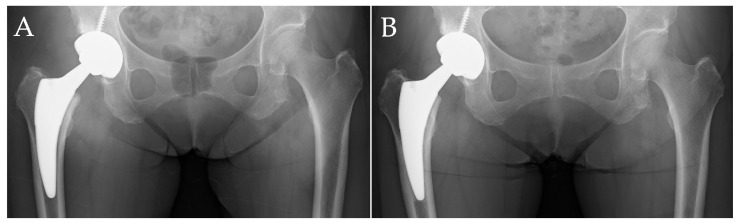
A representative case without evidence of stress shielding. Case of a 76-year-old female patient. (**A**) Anteroposterior radiograph of the hip immediately after surgery; (**B**) anteroposterior radiograph one year postoperatively. At one year postoperatively, no significant cortical bone thinning was observed, and there were no definitive indications of stress shielding.

Figure 2 shows images of a case without evidence of stress shielding, taken immediately after surgery and at one year postoperatively. The patient was a 76-year-old woman with a comorbidity of systemic lupus erythematosus and osteoporosis induced due to glucocorticoid use. Osteoporosis treatment involved the use of denosumab. The THA was performed using a Fitmore stem and Continuum cup (Zimmer, Warsaw, IN, USA). Even in the images taken one year postoperatively, there were no findings of proximal femoral bone atrophy, and Engh’s classification was Grade 0 (Figure 2B).

**Figure 3:**
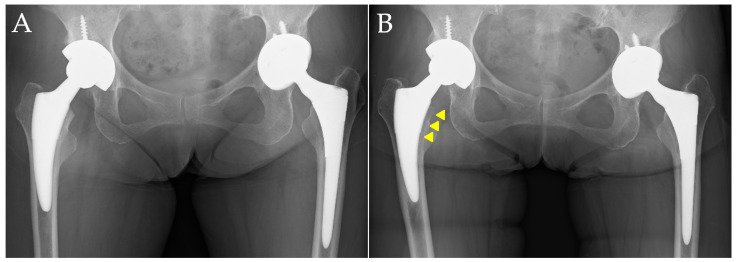
A representative case of advanced stress shielding. Case of a 73-year-old female patient. (**A**) Anteroposterior radiograph of the hip immediately after surgery; (**B**) anteroposterior radiograph one year postoperatively. The arrowheads indicate cortical bone thinning due to stress shielding, with progression of thinning observed up to the midsection of the stem.

In contrast, Figure 3 presents images of a case with evidence of stress shielding, taken immediately after surgery and at one year postoperatively. This patient was a 73-year-old woman without a diagnosis or treatment for osteoporosis. The femoral stem and acetabular cup used were also the Fitmore stem and Continuum cup (Zimmer, Warsaw, IN, USA). In the images taken one year postoperatively, significant findings of proximal femoral bone atrophy were observed, with an Engh’s classification of Grade 3 (Figure 3B).

## 4. Discussion

In this study, osteoporosis treatment intervention significantly reduced the occurrence of stress shielding. The preoperative osteoporosis treatment intervention rate was 28.9%. While there was no significant difference in clinical scores one year after surgery, it is suggested that such interventions may influence long-term outcomes. Preoperative bone mineral density assessment and osteoporosis treatment interventions are considered beneficial for the prevention of bone loss after THA.

Osteoporosis is a metabolic bone disease characterized by loss of bone strength, leading to fragility fractures [21]. The mechanism of osteoporosis involves an imbalance in bone remodeling, where osteoclast-mediated bone resorption exceeds osteoblast-mediated bone formation [22].

Bisphosphonates, a class of drugs used to treat osteoporosis, work by promoting bone mineralization and inhibiting osteoclast activity. Zoledronic acid, a specific bisphosphonate, is reported to have potential efficacy in preventing osteoporotic fractures and enhancing periprosthetic bone quality [23]. Furthermore, studies have demonstrated that intravenous administration of zoledronic acid can significantly reduce periprosthetic bone mineral loss following THA [24,25].

Denosumab, a human monoclonal antibody, is approved in numerous countries for the treatment of postmenopausal osteoporosis in women at high risk for fractures. Denosumab works by binding to the receptor activator of nuclear factor κB ligand, a cytokine essential for osteoclast formation, thereby inhibiting its interaction with the receptor activator of nuclear factor κB receptor on osteoclasts and their precursors [26]. Compared to alendronate, denosumab more effectively inhibits bone remodeling and reduces bone porosity, so in addition to suppressing systemic bone loss, it also suppresses bone destruction in rheumatoid arthritis [27] and may even reduce bone loss around artificial joints [28]. Denosumab has been reported to prevent bone loss and implant loosening following THA [16,17].

Activated vitamin D preparations enhance calcium absorption in the intestinal tract and improve calcium metabolism. Eldecalcitol, an activated vitamin D preparation, retains the calcium metabolism-improving effects of conventional activated vitamin D drugs while offering enhanced benefits for bone metabolism, making it a potential agent for fracture prevention. A comparative study between alphacalcidol and eldecalcitol demonstrated that the rate of bone mineral density (BMD) increase in both the lumbar spine and proximal femur was significantly higher in the eldecalcitol group [29].

In this study, the osteoporosis treatment intervention group exhibited a significantly lower incidence of postoperative stress shielding, although no impact was observed on postoperative clinical scores. According to the Bombelli classification, atrophic changes are more common in elderly women and individuals with acetabular dysplasia, which has been reported as a factor contributing to poor prognosis following THA [30,31]. The results of this study showed that the prior use of osteoporosis medication did not affect the degree of osteophyte formation in OA. Preoperative reductions in bone quality and BMD have been reported as risk factors for the development of stress shielding [20,32,33]. Previous studies have also reported that the continuous use of osteoporosis treatment drugs reduces bone resorption markers and may improve the long-term outcomes of artificial hip joint surgery [34,35]. The findings of this study align with previous reports. Osteoporosis treatment has been shown to lower revision rates after THA by reducing bone resorption markers and inhibiting osteoclast activity, suggesting that preoperative osteoporosis treatment intervention may have helped prevent early stress shielding.

This study has several limitations. First, this study is a retrospective analysis with a small sample size. A prospective study with a larger number of cases will be necessary in the future. Second, osteoporosis treatments were not standardized across the participants. The inhibitory effects of stress shielding among different medications were not evaluated due to the small number of cases and the uneven distribution of cases across the various drugs used. Thirdly, one limitation of this study is the lack of bone mineral density evaluation in the assessment of osteoporosis. Lastly, the short follow-up period of only one year represents another limitation. To gain more comprehensive insights, prospective studies with larger populations, longer-term follow-up, and an evaluation of complication rates are necessary.

## 5. Conclusions

In this study, the preoperative osteoporosis treatment intervention rate was 28.9%. Osteoporosis treatment intervention significantly reduced the incidence of stress shielding. Although no significant differences were observed in clinical scores one year postoperatively, it was suggested that preventing bone atrophy-related pain due to stress shielding and reducing fracture risk could positively impact the long-term outcomes. Preoperative bone density assessment and osteoporosis treatment interventions were considered beneficial for improving the clinical outcomes of THA.

## Figures and Tables

**Table 1:** Baseline patients’ characteristics.

Clinical Parameter	Treatment Group (n = 31)	Non-Treatment Group (n = 76)	*p*-Value
mean age (years)	74.9 ± 7.1	71.8 ± 6.8	*p* = 0.033
BMI (kg/m^2^)	24.9 ± 4.5	24.2 ± 3.8	*p* = 0.459
bone metabolism markers			
BAP (μg/L)	12 ± 4.3	17.7 ± 6.3	*p* < 0.0001
total P1NP (ng/mL)	44.6 ± 21.6	75.2 ± 25.1	*p* < 0.0001
TRACP-5b (mU/dL)	384.0 ± 200.4	586.6 ± 174.6	*p* < 0.0001
Bombelli classification (hips)			
atrophic type	17	33	*p* = 0.129
normotrophic type	19	56	
hypertrophic type	0	5	

Data are shown as the mean ± standard deviation; *p*-values of < 0.05 are considered significant by the Student’s *t*-test and Chi-squared test difference.

**Table 2:** Comparison of stress shielding and clinical scores one year after THA.

	Treatment Group (n = 31)	Non-Treatment Group (n = 76)	*p*-Value
Stress shielding (hips)			
Grade 0	12	6	0.001
Grade 1	10	28	
Grade 2	12	46	
Grade 3	2	11	
Grade 4	0	3	
Postoperative scores			
JOA	77.9 ± 10.3	81.6 ± 11.0	0.087
HHS	81.5 ± 9.0	84 ± 11.1	0.246
JHEQ	50.5 ± 14.5	55.2 ± 17.2	0.149

Data are shown as the mean ± standard deviation; *p*-values of < 0.05 are considered significant by the Student’s *t*-test and Chi-squared test difference. JOA means Japanese Orthopaedic Association; HHS means Harris hip score; JHEQ means Japanese Hip Society Hip Joint Evaluation Questionnaire.

## Data Availability

The data that support the findings of this study are available upon request from the corresponding author.

## Abbreviations

The following abbreviations are used in this manuscript:

THA	total hip arthroplasty
HOA	hip osteoarthritis
JOA	Japanese Orthopaedic Association
JHEQ	Japanese Hip Society Evaluation Questionnaire
HHS	Harris Hip Score
